# The EyeHarp: A Gaze-Controlled Digital Musical Instrument

**DOI:** 10.3389/fpsyg.2016.00906

**Published:** 2016-06-21

**Authors:** Zacharias Vamvakousis, Rafael Ramirez

**Affiliations:** Music Technology Group, Department of Information and Communication Technologies, Universitat Pompeu FabraBarcelona, Spain

**Keywords:** music performance, gaze interaction, digital musical instrument, accessible interfaces, disabilities

## Abstract

We present and evaluate the EyeHarp, a new gaze-controlled Digital Musical Instrument, which aims to enable people with severe motor disabilities to learn, perform, and compose music using only their gaze as control mechanism. It consists of (1) a step-sequencer layer, which serves for constructing chords/arpeggios, and (2) a melody layer, for playing melodies and changing the chords/arpeggios. We have conducted a pilot evaluation of the EyeHarp involving 39 participants with no disabilities from both a performer and an audience perspective. In the first case, eight people with normal vision and no motor disability participated in a music-playing session in which both quantitative and qualitative data were collected. In the second case 31 people qualitatively evaluated the EyeHarp in a concert setting consisting of two parts: a solo performance part, and an ensemble (EyeHarp, two guitars, and flute) performance part. The obtained results indicate that, similarly to traditional music instruments, the proposed digital musical instrument has a steep learning curve, and allows to produce expressive performances both from the performer and audience perspective.

## 1. Introduction

Music performance and learning to play a musical instrument have been showed to provide several benefits for acquiring non-musical skills (Coffman, 2002). For instance, musicians have an improved ability to hear speech in noisy backgrounds (Parbery-Clark et al., 2009), reduced age-related auditory degradation (Parbery-Clark et al., 2011), increased verbal and auditory memory (Chan et al., 1998; Ho et al., 2003), and enhanced auditory attention (Strait et al., 2010). Music instrument training is associated with neurostructural changes (Wan and Schlaug, 2010; Besson and Schön, 2012) both in children (Hyde et al., 2009) and adults (Bangert and Altenmüller, 2003). Motor brain regions are enlarged in musicians, when compared to non-musicians (Elbert et al., 1995). Gray matter volumes tend to be larger in musicians than in non-musicians for motor, auditory and visio-spatial brain regions (Gaser and Schlaug, 2003). Furthermore, gray mater density is greater in Broca's (language) area for trained musicians (Sluming et al., 2002). The corpus callosum, the fibers connecting the left and right hemispheres was found to be larger in musicians compared to non-musicians (Schlaug et al., 1995). Musicians' resistance to age-related neural decline is greater for musicians when compared with non-musicians (Pagnoni and Cekic, 2007). Early instrumental musical training seems to train attentional networks in the brain, as well as social and interpersonal skills. Children exposed to musical training show improvements in nonverbal memory, IQ, numeracy and spatial cognition (Neville et al., 2008). However, due to lack of fine motor skills, people with motor disabilities are often incapable of learning to play a musical instrument and thus, the benefits of music learning and performance are inaccessible to them. In this context, adaptive digital musical interfaces (ADMI) provide a possible alternative for allowing people with motor disabilities to enjoy music playing and its associated benefits.

The idea of implementing Adaptive Digital Musical Instruments (ADMI) for people with motor disabilities is not new. Depending on the type of motor disability, various ADMIs have been proposed. Kirk et al. (1994) presented the MidiGrid and the MidiCreator. The MidiCreator can be connected to a variety of sensors, such as an ultrasonic distance sensor or a pressure sensing foam. Through the midiGrid interface, the user can assign different music events to the messages sent by the MidiCreator. The system has been used in education and music therapy settings. Skoog is a low-cost pressure and deformation sensitive cube. It has served as a musical instrument for people with cerebral palsy^1^. Another example of tangible musical interface is TouchTone, proposed by Bhat (2010). It consists of 10 keys arranged in two rows. The arrangement and size of the buttons make the interface accessible to users with limited fine motor movements. Swingler (1998) introduced the SoundBeam. The input is provided by an ultrasonic distance sensor, that might be accompanied by buttons adapted to the needs of the user. The distance from the sensor along with the direction and speed of the part of the body that serves as input, are converted to midi data. Soundbeam is a commercial product. Webcamera-based low-cost systems are widely used (Winkler, 1997; Lamont et al., 2000; Stoykov and Corcos, 2006; Oliveros et al., 2011). Typically the screen is separated in distinct areas and when a movement is detected in each area, an event is triggered. All the above mentioned interfaces are designed for people that preserve a degree of limb movement. For people without adequate control of limb movements, an interface like the Magic Flute ^2^ might be appropriate. It is a head and breath controlled digital musical interface (DMI). The volume is controlled by blowing in a mouthpiece and the pitch by moving the mouthpiece up/down with the mouth.

In more severe cases of motor disabilities, such as people with locked-in syndrome (LIS), none of the mentioned interfaces is appropriate. LIS is a condition in which a patient is conscious but not able to move or communicate verbally due to complete paralysis of nearly all voluntary muscles in the body except the muscles which control the eyes (Bauer et al., 1979; Smith and Delargy, 2005). In such cases communication through eye-tracking technology might be the only alternative.

In eye-tracking-based (gaze-controlled) applications, gaze data might be used alone or in combination with other input methods, such as head, limb, or breath-controlled buttons. Blinking (closing both eyes) or winking (closing just one eye) might also be used as input. In this case, usually the gaze coordinates are used for pointing, and any other input is used for triggering actions. In case the gaze input is used alone, as the eye movements are often non-intentional, gaze information must be interpreted carefully to avoid unwanted responses to user actions. This is described as the “Midas Touch” problem. The most common gaze selection methods that intend to handle the Midas touch problem are: (i) screen button introduced by Ohno (1998) and (ii) Dwell time introduced by Jacob (1991).

In the case of screen button method, each target is separated in the command name area and the selection area. Selections are made only when a fixation is detected in the selection area. An extension of the screen button method is the pEYE method introduced by Huckauf and Urbina (2008), in which the “slices” of the “pEYE” are screen buttons. The command name areas of the buttons are placed at the interior of the pie, and the selection areas are placed at the perimeter.

In the case of the dwell time method, when a fixation lasts for more than a given time period (typically about 1 s), a selection is made. The spatial accuracy of eye trackers is usually limited, not allowing the selection of small targets. Dwell time selection method is often combined with magnification methods in order to increase accuracy in gaze pointing and selection (e.g., Lankford, 2000; McGuffin and Balakrishnan, 2002; Zhai et al., 2003; Ashmore and Duchowski, 2005; Fono and Vertegaal, 2005; Hegner and Skovsgaard, 2008). In that case the selection is normally divided into two stages: (i) selecting the area to be magnified and (ii) selecting a target within that area.

An extensive review of eye-controlled music performance systems was recently made by Hornof (2014). Some of these installations do not aim to resemble traditional musical instruments: they could be described as sonifications of eye movements and they are not designed for playing melodies. Here we will only refer to the approaches that provide the possibility of playing separate notes. Duet for eyes^3^ was a performance including performers with and without disabilities. The Grid software ^4^, using dwell-time selection method and controlled by a Tobii eye tracker ^5^, was used to trigger preselected sounds. In a more recent project, called “eye play the piano” ^6^, by the University of Tsukuba and FOVE eye tracking virtual reality headset^7^, people with disabilities were able to trigger notes or chords of a piano, assigned to buttons on the screen. Blinking was used as a selection method. In the mentioned gaze-controlled setups dwell-time and blinking selection methods are used for triggering musical events. Dwell-time does not allow triggering events in tempo, as it implies a big latency, while blinking selection requires two actions in order to trigger a single event: (i) focusing on a target and (ii) blinking. Moreover, none of the mentioned systems allows the control of more expressive musical features, such as loudness.

In this study we propose an interface in which only the gaze is used as input and which allows a similar interaction and expressiveness as traditional musical instruments. The EyeHarp, using the screen button gaze selection method, allows the control of chords, arpeggios, melody, and loudness using only the gaze as input. Eight people with medium to advanced musical skills took part in an experimental session in which the usability of the EyeHarp was quantitatively and qualitatively studied from the perspective of the performer. Additionally, the EyeHarp was evaluated by 31 participants from the perspective of the audience in a concert setting which consisted of two parts: a solo performance and an ensemble performance (EyeHarp, two guitars, and flute).

## 2. Materials and methods

### 2.1. The EyeHarp

The EyeHarp allows the user to control pitch, timing and dynamics of a melody, as well as chords and arpeggios in a performance. The EyeHarp interface consists of two layers: the Step Sequencer layer and the Melody layer. In the Step Sequencer layer chords and arpeggios can be constructed and in the melody layer these can be controlled and a melody can be played. The number of available note buttons can be adapted according to the accuracy of the eye tracker and the expertise of the performer. The user can switch between the two layers through a dwell-time activated button.

The EyeHarp is implemented using openFrameworks open source C++ toolkit^8^. It has a built-in analog software synthesizer and it also works as a midi device, controlling any external software synthesizer. The EyeHarp is currently an open-source software^9^ that runs in windows 7 or later operating systems. Currently two commercial eye-trackers are supported: the Eyetribe^10^ and Tobii PCEye^11^. The non-commercial open-source ITU Gazetracker^12^ is also supported. In all three cases the EyeHarp receives through a server the raw gaze data. Fixation detection and smoothing algorithms are incorporated in the EyeHarp software. This allows a consistent behavior of the system when different eye trackers are used.

The interface is diatonic and by default tuned to the C major scale. Nevertheless, through a configuration menu it can be tuned to any possible scale. Only the basic functionality of the EyeHarp interface will be described here. A more detailed overview of the more advanced features of the interface was presented by Vamvakousis and Ramirez (2011).

#### 2.1.1. The step sequencer layer

**Figure 1:**
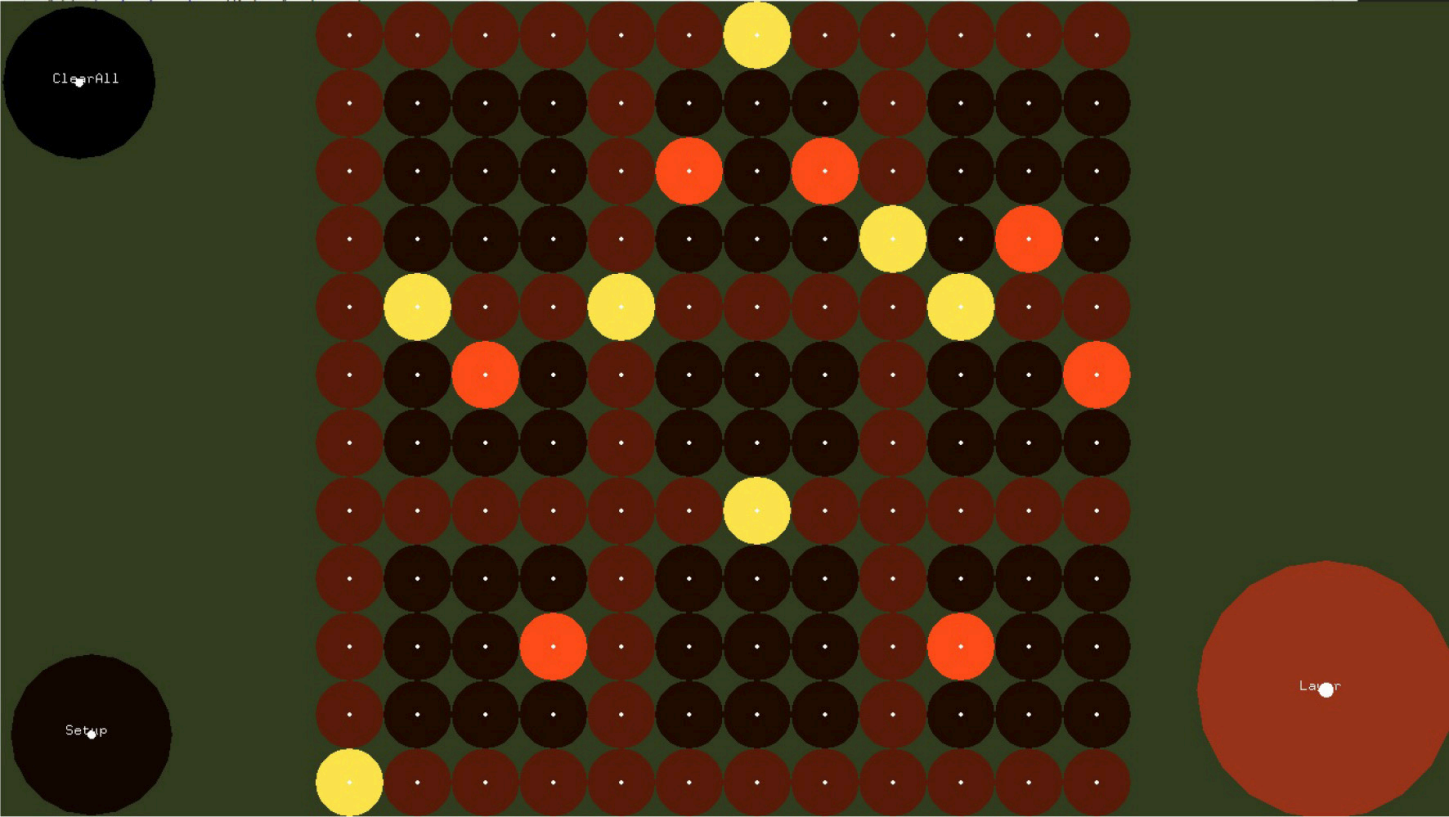
**The Step Sequencer Layer**. In this layer the user can construct arpeggios and chords which are controlled in the Melody Layer. Buttons in the same row correspond to notes with same pitch, while buttons in the same column correspond to simultaneous notes. If the selected chord in the melody layer is C major, buttons from bottom to top correspond to notes C4, D4, E4, etc. Notes are triggered from let to right, starting with the left most column. Dwell-time selection method is used, i.e., users focus at each button for about 700 ms in order to select or release a button.

Figure 1 shows the Step Sequencer layer. A step sequencer is an interface for constructing loops. It consists of a grid of buttons where the vertical dimension of the grid corresponds to pitch and the horizontal dimension corresponds to the temporal position in the loop. At the beginning of the loop, the selected notes of the first column sound simultaneously, followed by the selected notes of the second columns, and so on. After the notes of the last column are played, the loop starts over. The time interval between the activation of two consecutive columns is constant and depends on the set tempo.

**Figure 2:**
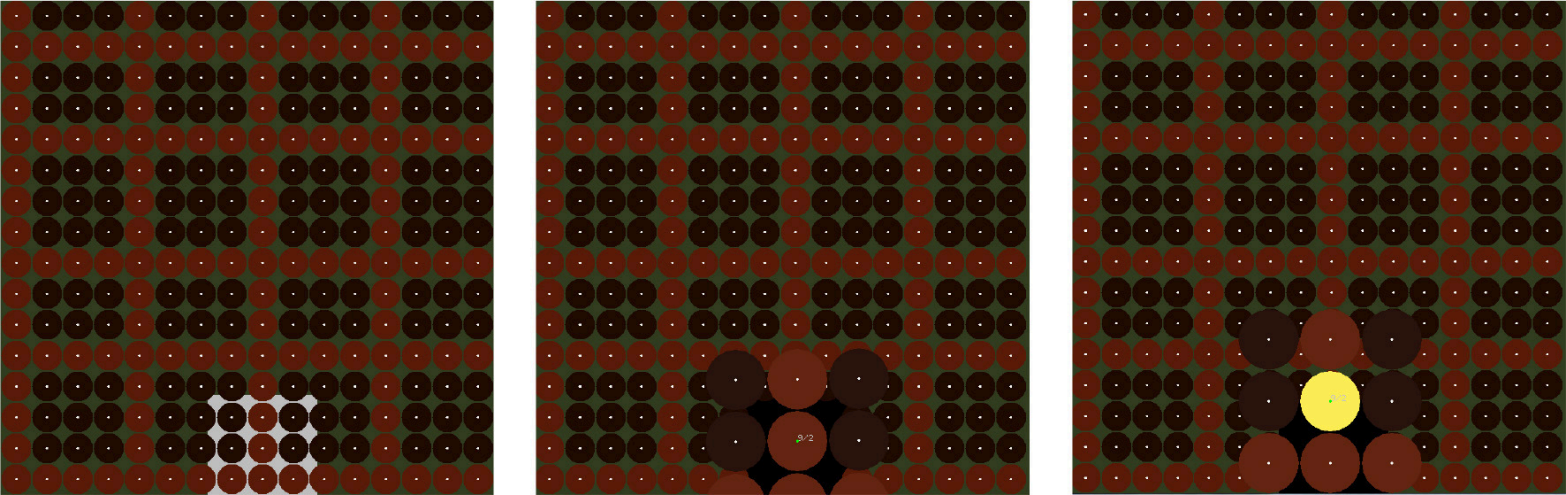
**The Magnification method for improving spatial selection accuracy**. If the magnified area appears outside the screen, it smoothly moves inside.

**Figure 3:**
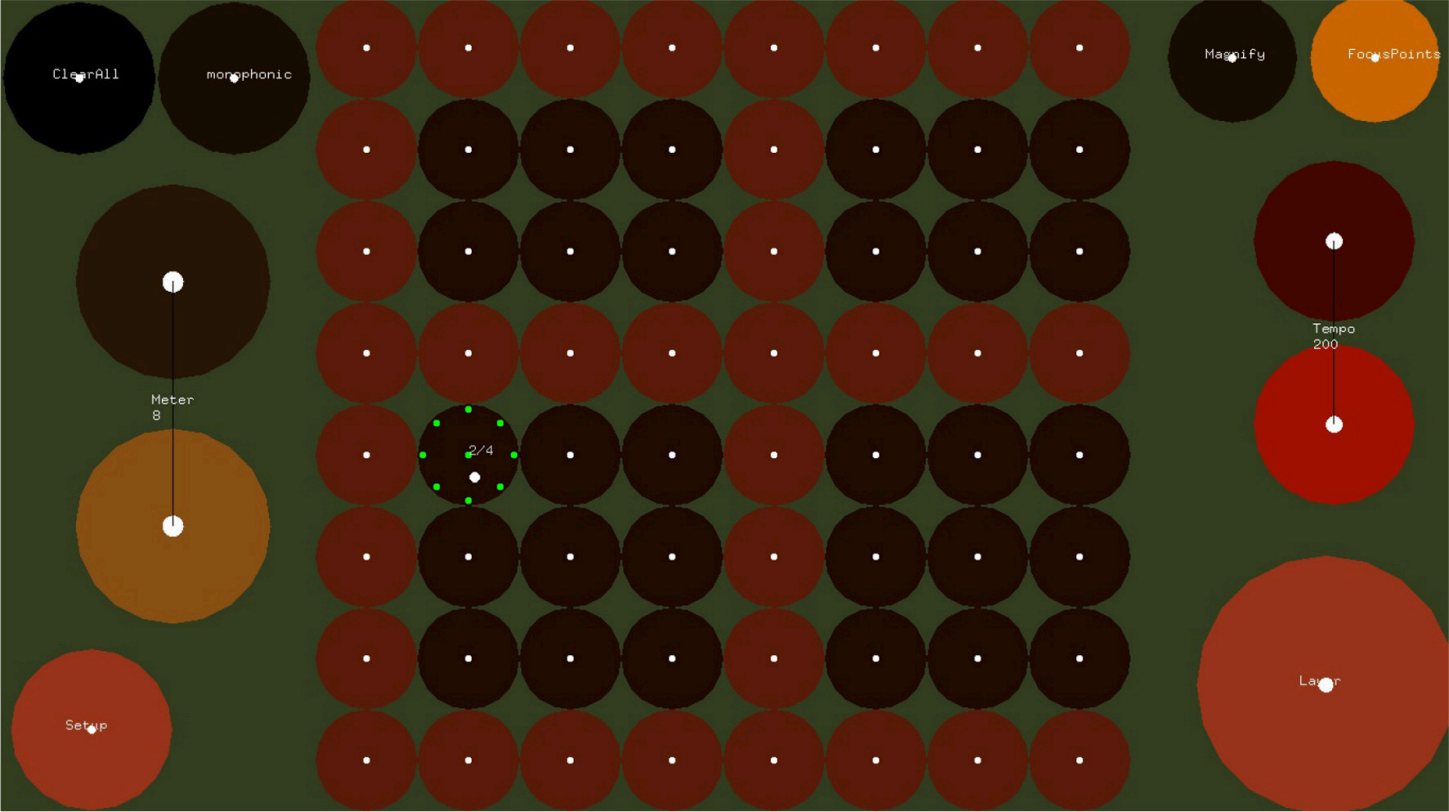
**The Gaze Feedback method for improving spatial selection accuracy**.

In order to select a button of the step sequencer, dwell-time selection is applied. The default dwell time value of the EyeHarp interface is 700 ms. The buttons are cyclic with a small focus point at the center. The focus point helps the users to focus their gaze at a point in the center of the target thereby improving the accuracy of the tracking data(Kumar et al., 2008). The Step Sequencer layer includes two methods for improving the spatial accuracy of the eye-tracker. In the first method the gaze point appears on the screen along with additional focus points at the perimeter of the buttons. This helps the user correct the offset caused by poor tracking. A similar method was proposed by Kumar et al. (2008). In the second case, when a fixation is detected and the dwell time period is reached, the buttons of the step sequencer that are placed within a square region -centered at the fixated point and covering the 20% of the sequencer area- are magnified by a factor of 2. The user can then select one of the magnified buttons. By looking outside the magnified area, all buttons return to their normal size and position. Figures 2, 3 demonstrates the two described methods. Note that in case of the magnification method, as the buttons expand, they might come out the screen. In that case all magnified buttons smoothly move up or down in order to appear inside the screen.

A number of control buttons (e.g., for changing the meter or tempo, clearing the selected notes, switching between layers) are provided and may be selected using dwell time selection method (see Figure 3).

The Step Sequencer Layer is a layer for constructing arpeggios, whose harmony is controlled in the melody layer. The note that corresponds to bottom row of the EyeHarp's Step Sequencer is determined by the base note of the selected chord in the melody later. The notes corresponding to the other rows in the step sequencer are mapped to the consecutive notes. For example, if the EyeHarp is tuned to the C major scale and the selected chord in the Melody Layer is the tonic (C major), the buttons of the first row correspond to the note *c* in the 3rd octave. The buttons in the second row correspond to the note *d* and so on. In case the selected chord is the dominant (G Major), the first row is mapped to the note *g* in the 3rd octave, the second to *a* and so on. In **Figure 7** there are examples of exercises performed with the EyeHarp. The repetitive arpeggios in the bass clef are constructed in the Step Sequencer Layer. But the actual notes played depend on the selected chord in the Melody Layer. For example, in task 4, when the tonal chord is selected (C Major), the arpeggio consists of the the notes “c-e-g-e.” When in bar 4 the dominant chord (G Major) is selected the notes of the arpeggio change to “g-b-d-b.”

#### 2.1.2. The melody layer

**Figure 4:**
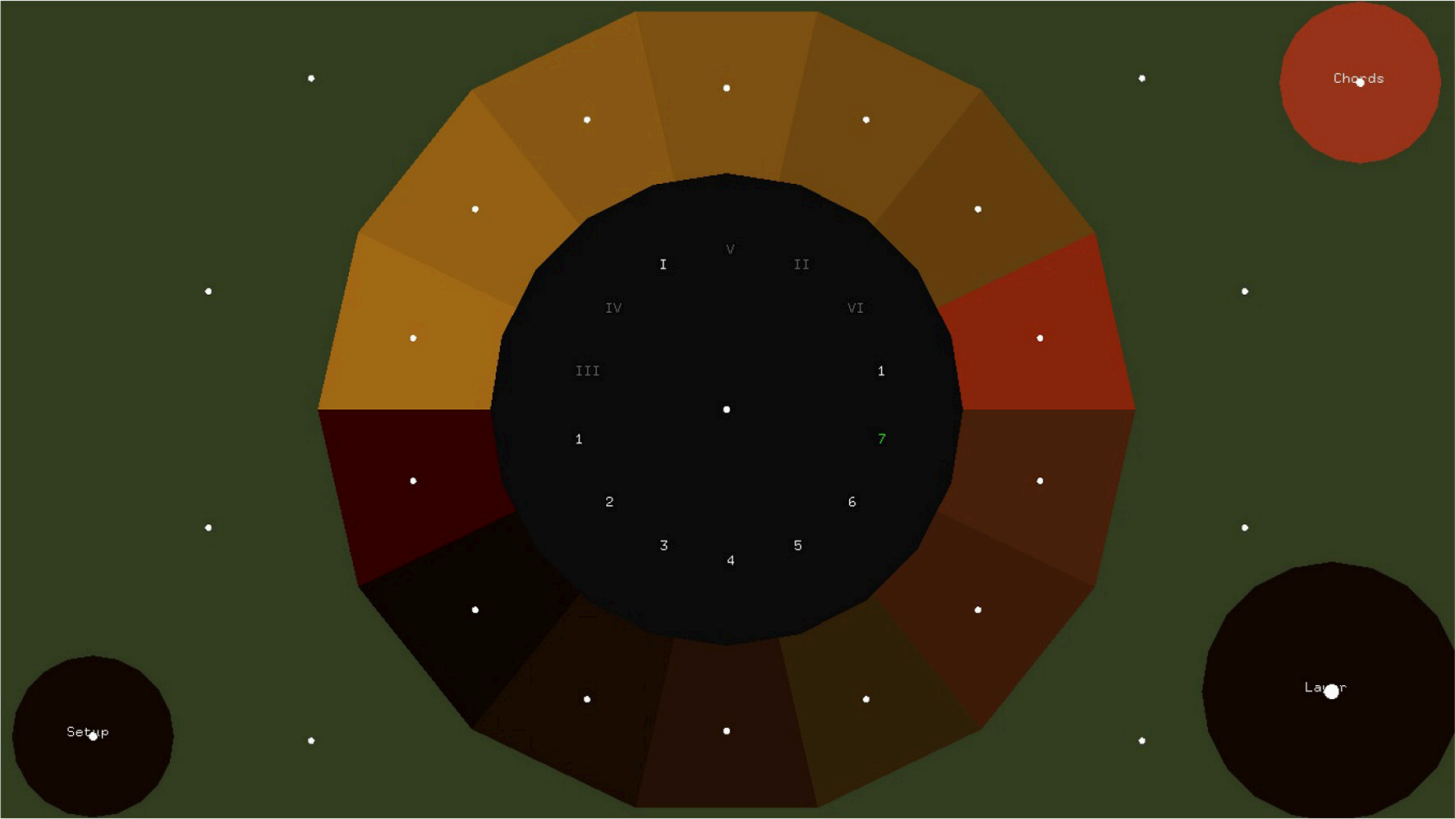
**The Melody Layer in which the user plays melodies and changes the chords/arpeggios constructed in the step sequencer layer**. The melody layer buttons are placed over the perimeter of a circle, leaving the area in the center inactive.

**Figure 5:**
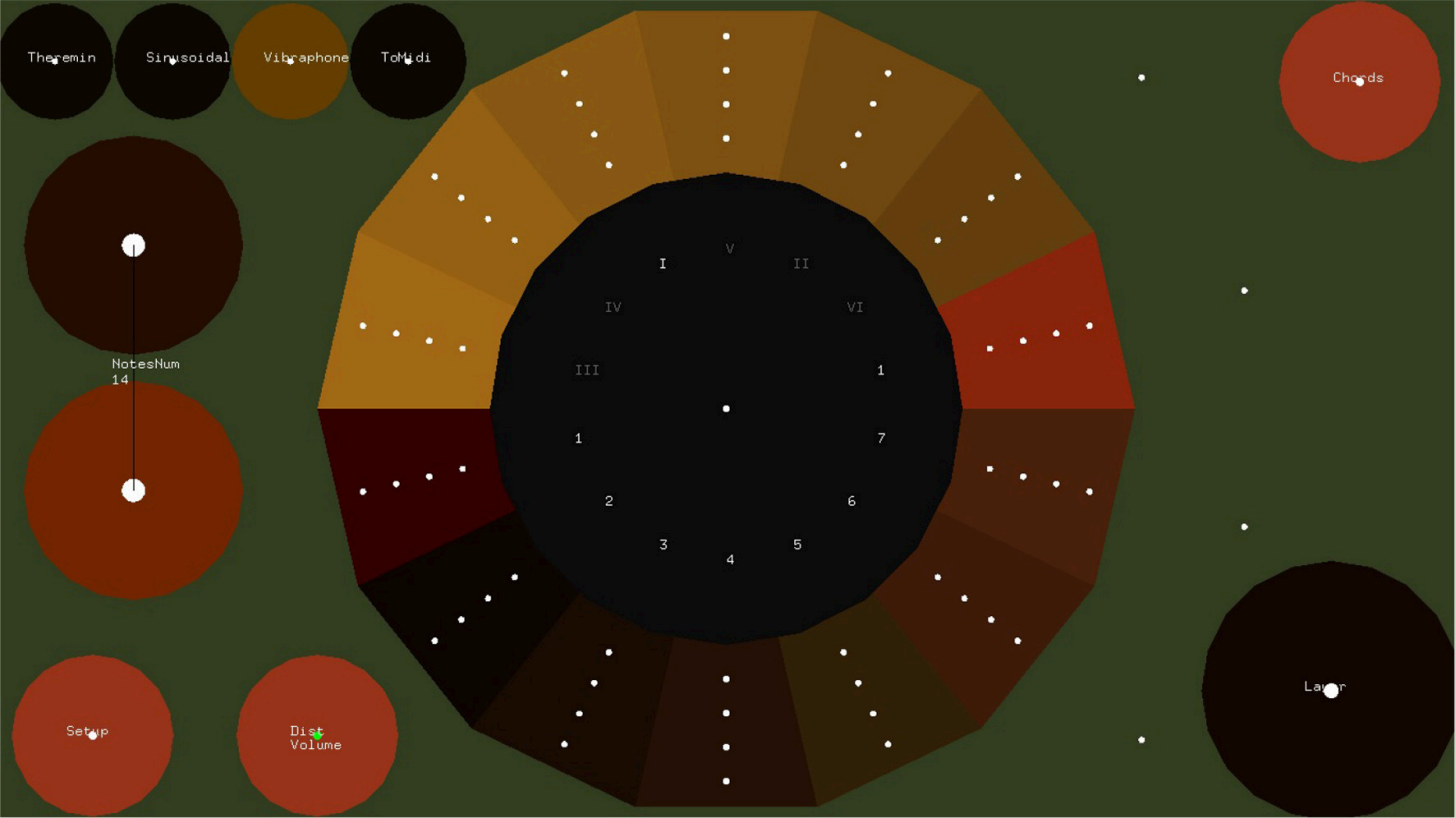
**The control buttons**. By activating through dwell-time the “Setup” button, the user can change various parameters of the interface including “dynamics” button which allows to map the distance from the center of the “pie” to the loudness of the performed note.

The Melody layer (Figure 4) is based on pie menus. A pie menu is made of several “pie slices.” Each slice consists of an inactive area in the center and the selection area at the perimeter of the circle. The idea of using pie menus in gaze interaction was introduced by Huckauf and Urbina (2008) in a typing and a desktop-navigation interface. The idea of the pEYE layout is appealing for playing melodies because clicking is not necessary for making a selection. Once the pointer enters in the selection area at the perimeter of the pie, a command is triggered. The slices of the pie menu of the Melody layer can be thought as screen buttons (as introduced by Ohno, 1998). The command name area is a number for each note and a Latin number for each chord. The selection area is placed at the perimeter of the pie. At the center of the selection area of each note, a focus point appears. Optionally as shown in Figure 5, multiple focus points appear in the selection area of each slice. Outer focus points correspond to high loudness and vibrato, while inner points correspond to lower loudness and vibrato.

If the set scale is C major, c in the 4th octave is placed at 180°. The scale then goes up counterclockwise. As a default option the pie comes with 14 slices, but the number of slices can be adapted though the setup menu. If the setup button is pressed in the melody layer, a number of configuration buttons appear as shown in Figure 5. Two repeat buttons on the left can be used for adjusting the number of notes in the pie. Through four radio buttons on the top the user can select between three preset sounds of the EyeHarp internal synthesizer, or select the midi out option. In that case the interface is sending midi messages to an external synthesizer through the LoopBe virtual midi port^13^.

If the “chords” button is active, the last six notes of the pie are replaced by six chords. These buttons control the harmony of the arpeggio constructed in the Step Sequencer layer as explained in Section 2.1.1. In order to play a note or change the chord, the user can either look directly at the selection area of the note/chord or -in case there is a big distance on the screen between two consecutive notes- he can focus on the command name area before focusing on the selection area. This is expected to improve the spatial and temporal accuracy, as Fitt's law also applies to gaze interaction as shown by Miniotas (2000).

In order to release a note, the user has to look at any place outside the pie. For that reason some fixation points are placed outside the pie. When a fixation is detected at the selection area of a note the note sounds and a button appears at the center of the pie. This allows the user to repeat the same note twice. If a fixation is detected inside the button's area, the same note sounds again. If a fixation is detected elsewhere inside the inner (neutral) area, the “repeat” button disappears.

### 2.2. Evaluation

O'Modhrain (2011) proposed that a DMI can be evaluated from the perspective of (i) the audience, (ii) the performer, (iii) the designer, and (iv) the manufacturer. In this evaluation process we evaluate the proposed interface from the perspective of the audience and the performer.

#### 2.2.1. Audience perspective

Transparency describes the level to which a performer or spectator can understand the relationship between the input (gesture) and output (sound). According to Hunt et al. (2002) and Arfib et al. (2005), an instrument's capability for expressive performance is positively correlated to its degree of transparency, i.e., how clear is the mapping between the gestures of the performer and the sound produced by the instrument. Unlike traditional musical instruments, in DMIs the way the performer's gestures produce sound might not be physically evident to the audience. Schloss (2002) suggests that the lack of an obvious connection between cause and effect dramatically affects the way a performance is perceived by the audience. According to Schloss, providing visual cues that aim to reestablish the connection between cause and effect is a key component in making a DMI performance convincing and effective.

Reeves et al. (2005) proposed an evaluation of DMIs based on audiences perception of the relationship between input manipulations and audio output. They characterize a performance with low input and output comprehension as “secretive,” one with low input and high output comprehension as “magical,” one with high input and low output as “suspenseful,” and one with high input and output as “expressive.” Barbosa and Calegario (2012) extended Reeves's classification and proposed five different aspects to be considered in building the “interaction model” of a DMI: (i) The cause comprehension refers to how clear the available input gestures are. (ii) The effect comprehension refers to how clear the controlled parameters are. (iii) The mapping comprehension refers to how clear is the relation between user's actions and the resulting sound. (iv) The intention comprehension refers to what degree the system allows the user to express his musical intentions. (v) The error comprehension refers to whether the possible errors in the performance were noticeable.

A concert was organized at the concert hall of Universitat Pompeu Fabra. The performer had been practicing the EyeHarp for a period of 10 weeks, playing three times a week. Every practice session lasted for ~20 min. The concert consisted of two parts. In the first part the EyeHarp player performed a piece composed by him for EyeHarp solo performance and in the second he performed along with two guitar players and a flute player in a jam session. One of the eyes of the performer was shown at the center of the screen and the coordinates of his gaze were visualized by a small cross. A recorded video of the performance^14^ was then shown to a group of 31 people, none of whom was familiar with the EyeHarp. All participants reported at least a basic level in playing a musical instrument. Before showing the video performance, the audience was informed that the EyeHarp is a Gaze-Controlled digital musical instrument that consists of two different layers allowing the user to construct chords and arpeggios, control the harmony and play melodies. After having watched the video, the participants responded to a questionnaire. The questionnaire included questions intended to identify the profile of the listener (age, sex, music education, familiarity with DMIs and eye tracking technology) and questions exploring the evaluation criteria proposed by Barbosa and Calegario (2012). All responses were given in the form of linear scale from 1 to 5. Thirty-one people (6 women) of average age 30.5 years (standard deviation 5.8) responded to the questionnaire. Procedures were positively evaluated by the Parc de Salut MAR - Clinical Research Ethics Committee, Barcelona, Spain, under the reference number: 2013/5459/I. Participants responded questions for six evaluation criteria:

- Cause comprehension: were the available input gestures clear? (1: not at all. 5: very clear)
- Effect comprehension: were the available control parameters clear? (1: not at all. 5: very clear)
- Mapping comprehension: was the connection between the input gestures and the control parameters clear? (1: not at all. 5: very clear)
- Intention comprehension: how well did the system allow the user to express his musical intentions? (1: not at all. 5: very well)
- Error comprehension: if there had been errors in the performance, would they have been noticeable? (1: not at all. 5: very noticeable)
- Enjoyment: how much did you enjoy the performance? (1: not at all. 5: a lot)

#### 2.2.2. Performer perspective

##### 2.2.2.1. Quantitative evaluation

The performer perspective evaluation was carried out with written informed consent from eight participants in accordance with the Declaration of Helsinki. Procedures were positively evaluated by the Parc de Salut MAR - Clinical Research Ethics Committee, Barcelona, Spain, under the reference number: 2013/5459/I. Participants (7 male, 1 female) with mean age of 34 years (SD 6.7) participated in a single-session quantitative evaluation task. All participants had some musical instrument playing experience. The quantitative evaluation consisted of a set of tasks using both the step sequencer and melody layer. Apart from one subject, no participant had previous experience with the EyeHarp DMI.

The Eyetribe low-cost commercial eye-tracker was used for acquiring the raw gaze data. Participants were comfortably seated at ~60 cm away from a 15.6 inches laptop screen placed at eyes level. All participants calibrated with nine calibration points and 800 ms of sample and transition time. All participants achieved a 5-star calibration quality in the Eyetribe calibration software (expected visual angle accuracy = 0.5°). A set of M-Audio AV40 self-amplified speakers were connected to the laptop audio output. The ASIO4ALL low latency driver was used, providing an audio output latency of 7 ms. The EyeHarp application was sending MIDI messages through loopBe1 virtual MIDI port to Reaper Digital Audio Workstation (DAW)^15^, running a piano sound module for the Step Sequencer layer and a clarinet sound module for the Melody layer. Gaze data were recorded in the EyeHarp application, whereas MIDI data were recorded in the Reaper DAW.

Step sequencer layer evaluation

**Figure 6:**
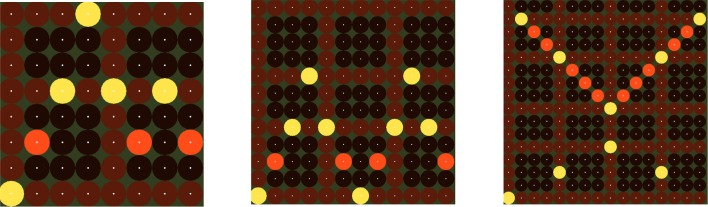
**The three evaluation tasks of the step sequencer layer**. The time required to complete each of the tasks was measured.

The step sequencer layer evaluation task consisted of constructing arpeggios with varying number of buttons in the step sequencer grid. All arpeggios were constructed three times. The first time the gaze pointer was hidden and no magnification method was applied (basic method). The second time the gaze pointer appeared along with additional focus point (gaze feedback method). The third time the gaze pointer was hidden and the described magnification method was applied (magnification method). In all cases when the gaze was detected inside a button, the fixation point of that button was turning green. Figure 6 shows the three different arpeggios the participants were asked to construct in each of the three tasks. In the first task the grid size was 8 × 8, in the second 12 × 12 and in the third 16 × 16. In all cases, the participants were asked to correct all possible mistakes. The time to complete each task was measured.

Melody layer evaluation

In a previous study Vamvakousis and Ramirez (2012) involving 10 subjects without motor disabilities, the temporal accuracy of the EyeHarp Melody layer was examined. The results can be summarized as follows:

- Notes tended to be played earlier (i.e., in advance). Two diametric distant buttons in the pEYE resulted in an average asynchrony of −46 ms, while two adjacent buttons resulted in −94 ms.
- The temporal accuracy of the participants improved with practice.
- The temporal variance value was 10 times higher when compared to the input from a computer keyboard.

**Figure 7:**
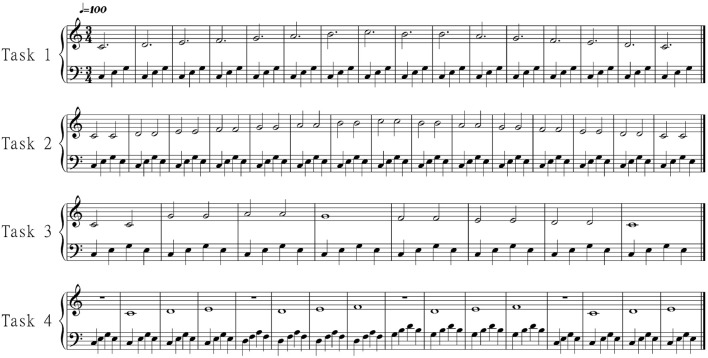
**The four evaluation tasks of the Melody layer**. The tasks are of increasing difficulty. In the last task the participants were asked to control both the harmony and the melody.

In the current evaluation, instead of examining the temporal performance of the interface, we examined the overall usability of the interface. Four different tasks of increasing difficulty were designed. Users practiced for about 2 min before recording three repetitions of each task. At the beginning of each task an arpeggio was constructed in the step sequencer layer that served as a metronome. Figure 7 shows the melodies the participants were asked to perform for each task: a scale in both directions, a scale with repeated notes, “twinkle twinkle little star,” and a music exercise with a melody and a chord progression.

##### 2.2.2.2. Qualitative evaluation

After the quantitative evaluation session participants filled in a questionnaire. Participants responded (in a linear scale from 1 to 5) to the following questions:

- How much previous practice and training does the performer need for performing with the instrument, when compared to a traditional musical instrument? (1: no practice required. 5: extensive practice required)
- How much control does the performer have on the musical output? (1: restricted (equivalent to a DJ). 5: extensive musical control that allows expressive performance.
- How much real-time feedback (e.g., visual, auditory) does the user receive from the system? (1: low feedback. 5: high, multimodal feedback)
- How tiring is it to play music with your eyes when compared to the hands? (1: not tiring at all. 5: very tiring)
- Is it hard to play in tempo with your eyes when compared to hands? (1: equally hard. 5: much harder.)
- Which approach between the magnification lens and the fixation points do you consider more user-friendly? (1: I prefer the fixation points. 5: I prefer the magnification lens)

All questions were verbally explained to the participants. If anything seemed unclear to the participants they were free to ask for questions, which were clarified orally. In the first question, it was orally clarified that by the phrase “performing with the instrument” it is meant to achieve some basic, but rewarding interaction with the instrument. By the response “1: no practice required” we refer to the practice required to achieve a rewarding performance in a gaming music interface, like the guitar hero of Microsoft Xbox. By the response “5: extensive practice”, we refer to the practice required to achieve a rewarding performance in a musical instrument that is considered to be difficult to learn, like the violin. Similarly, regarding the second question, it was clarified that by the response “5: extensive musical control that allows expressive performance” we refer to the control offered by an instrument like the violin. In question 4, it was orally clarified that users should respond “1: not tiring at all” if they consider it equally tiring as playing with the hands.

## 3. Results

### 3.1. Audience perspective

**Figure 8:**
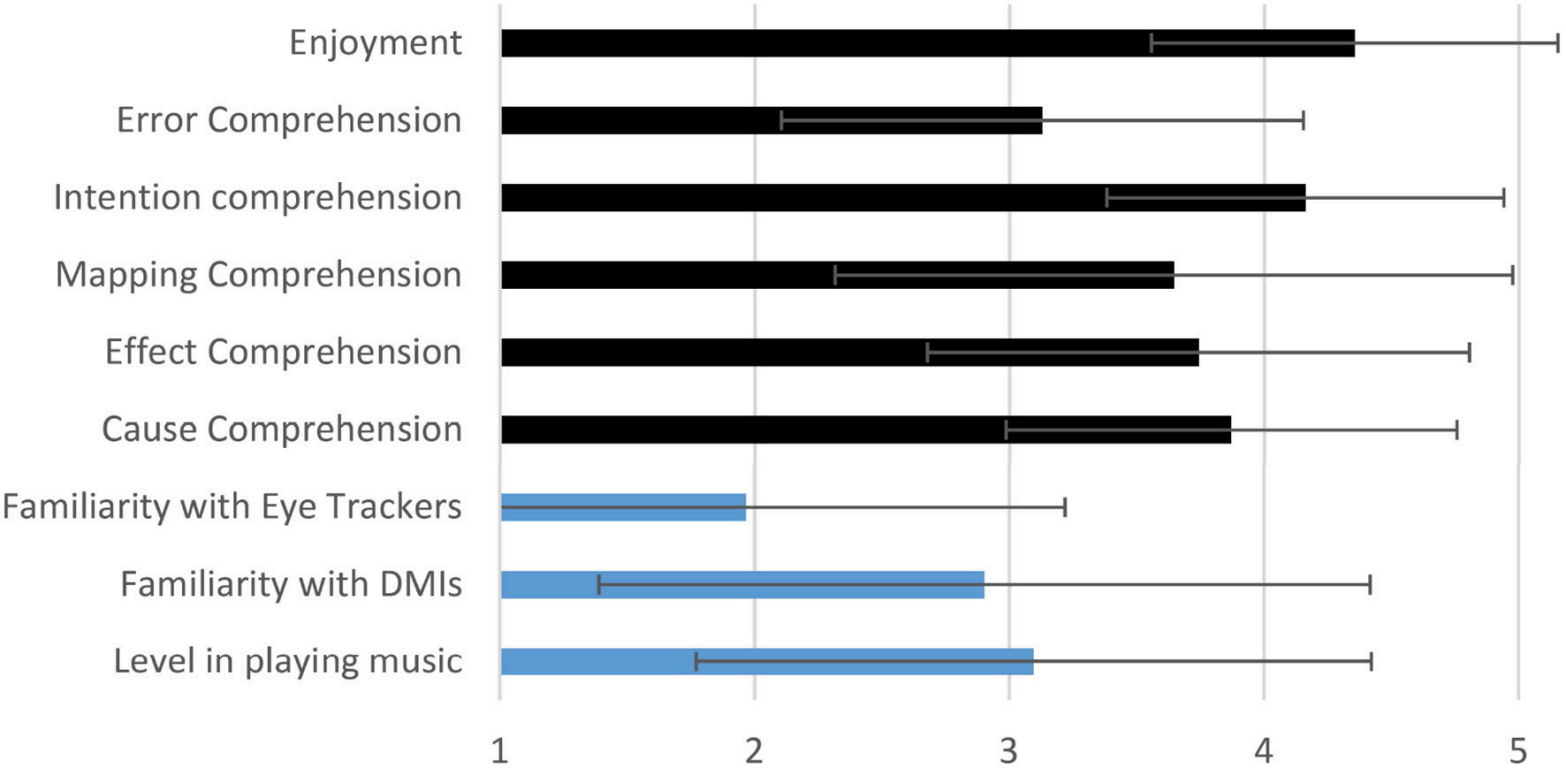
**Qualitative evaluation average results from the audience perspective across 31 participants**. In blue appear the questions related to the profile of the audience and in black the questions related to the evaluation of the DMI.

Figure 8 shows the average responses and the corresponding standard deviation across all participants. The responses of the audience can be summarized as follows: The available input gestures were clear (average = 3.9, σ = 0.87). The available control parameters were clear (average = 3.8, σ = 1.04). The connection between them was clear (average = 3.7, σ = 1.34). The system allowed the user express his musical intention very well (average = 4.2, σ = 0.76). Errors in the performance would have been noticeable (average = 3.1, σ = 1.06). Finally the audience enjoyed the performance a lot (average = 4.3, σ = 0.84).

### 3.2. Performer perspective

#### 3.2.1. Step sequencer layer

**Figure 9:**
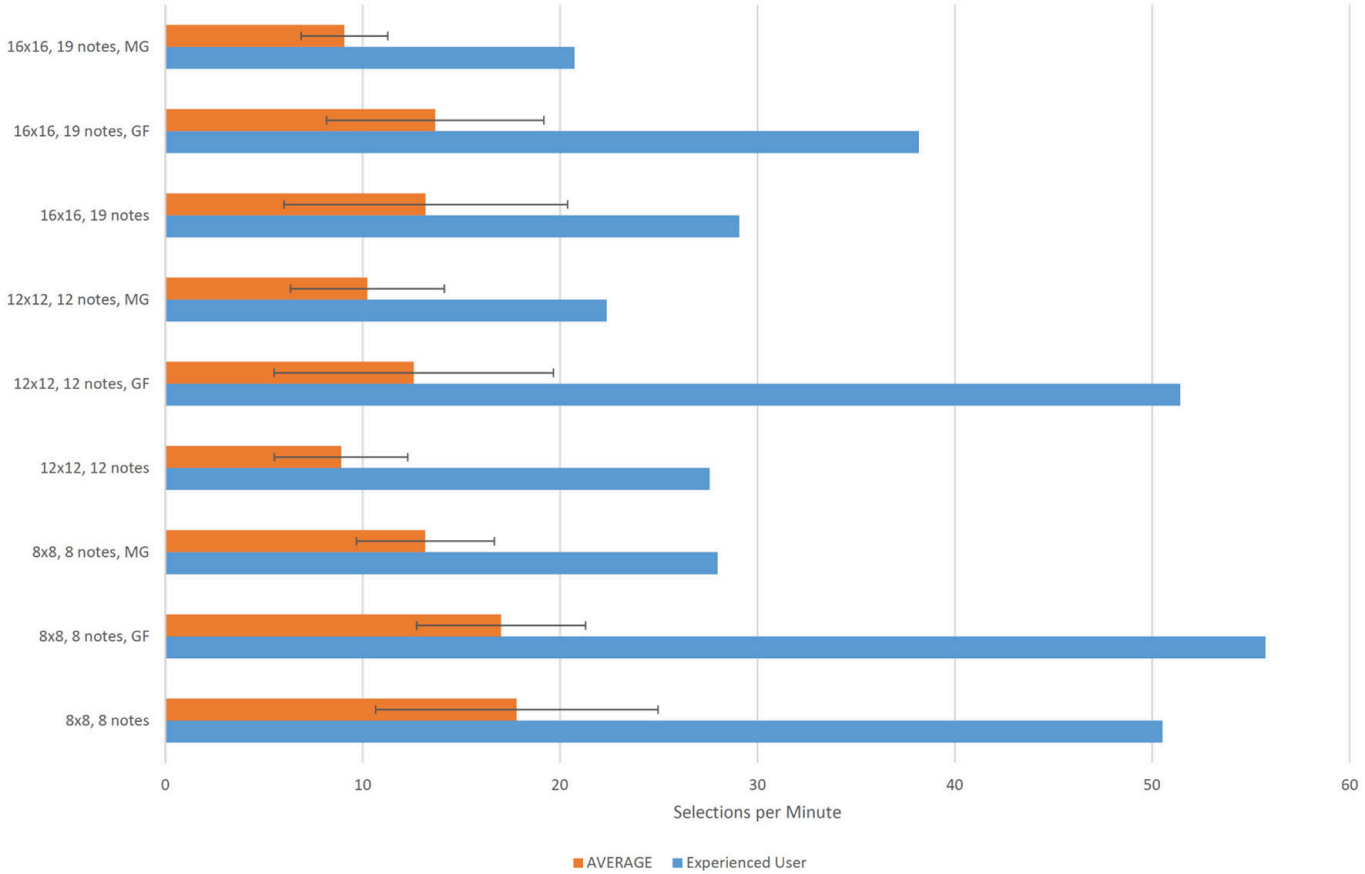
**Quantitative evaluation average results of the Step Sequencer Layer across seven novice users**. The results of one experienced user appear separately. The horizontal axes corresponds to the number of selections made per minute. For each user and task this value is computed by dividing the number of notes in the task by the time required to complete it. MG refers to the magnification method. GF refers to the Gaze Feedback method.

Figure 9 shows the average number of selections per minute across the seven participants with no previous experience with the interface, for each task. The results obtained by the experienced user (M32) are shown separately at the same graph. The average number of selections per minute value is computed by dividing the number of required selections in each task by the time to complete the task.

In all tasks the experienced user performed about 2 to 3 times faster than the average speed across the native users. The best average performance (selections per minute) in the case of the 12 × 12 and 16 × 16 grid was achieved with the gaze feedback method. In the case of the 8 × 8 grid task, it was achieved with the basic feedback method. The lowest standard deviation value was achieved in all tasks with the magnification method.

#### 3.2.2. Melody layer

**Figure 10:**
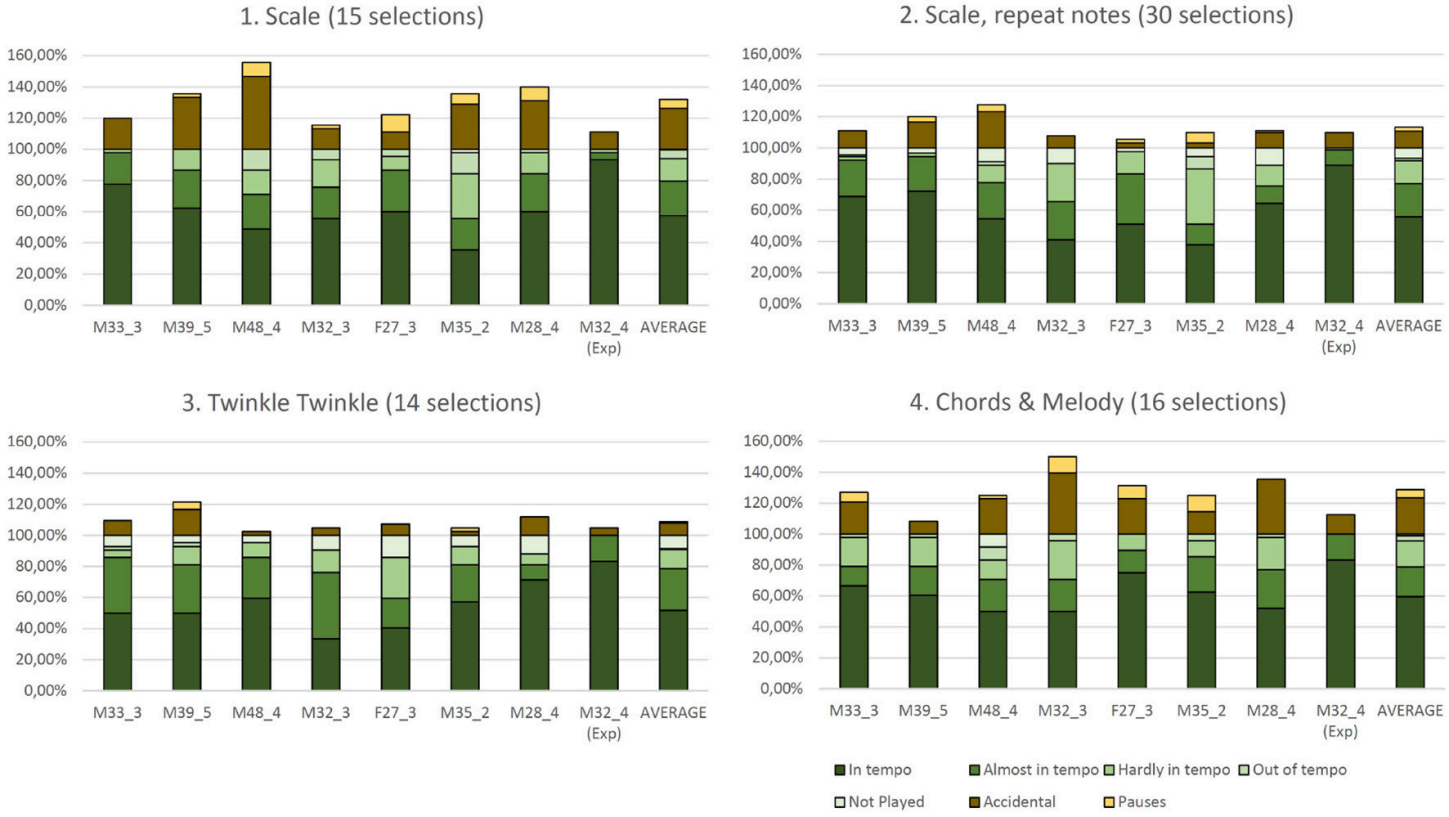
**Quantitative evaluation results of the Melody Layer for eight users for all four tasks**. The last column of the plot of each task shows the average performance across all seven users with no previous experience with the EyeHarp. Subject M28_4 is the only user with previous experience with the interface. The different shades of green correspond to the percentages related to the temporal accuracy of each task. The percentages are computed over the total number of selections required for each task. The darkest green corresponds to the percentage of notes played in tempo (accurate to within 1/16), followed by notes played almost in tempo (accurate to within 1/8), hardly in tempo (accurate to within 1/4), out of tempo and not played at all (omitted). All these values sum 100%. In dark brown appears the percentage of wrong or accidentally played notes and in bright brown appears the number of pauses in the task. Pauses refer to the number of times the users stopped during a task and waited till next bar in order to enter in tempo.

Figure 10 shows for each task and participant the percentage values of the notes played according to temporal accuracy. These values sum 100%, as they correspond to the temporal accuracy of played notes along with the omitted notes. In dark brown appears the percentage of accidentally played notes and in light brown appears the number of pauses made in each task. As pauses we refer to the cases where the participants stopped for one or more bars, in order to continue playing in tempo. The percentages are calculated by dividing the number of each value with the total number of selections that should be made in the task. The last column of each task corresponds to the average value across all participants, excluding the experienced participant. In Figure 10, the code number of each participants was given by considering their sex, age and level of playing music in a scale from 1 to 5 (1: not playing any instrument, 5: professional level). For example user M48_4 is a 48 year old man with semi-professional level in playing music.

In all tasks the experienced user played around 20% more notes in tempo than the novice users, performed less accidental notes and no pauses.

#### 3.2.3. Qualitative evaluation

**Figure 11:**
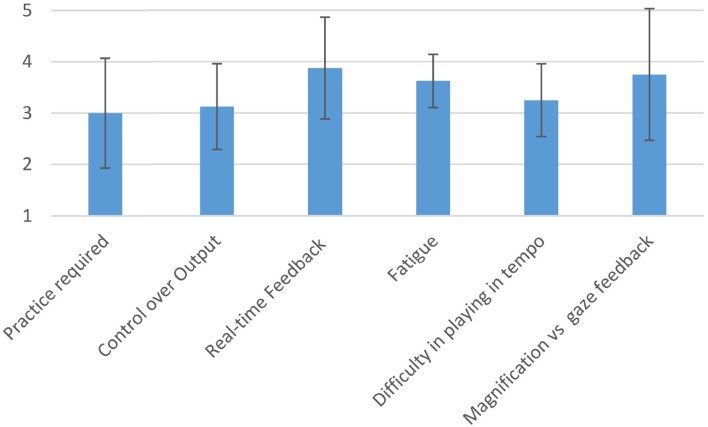
**Qualitative evaluation of the EyeHarp provided by the performers**. All answers were in a linear scale from 1 to 5. The average across all participants along with the standard deviation is given for each question.

Figure 11 shows the average and standard deviation of the responses of the participants in the performer's evaluation.

## 4. Discussion

In the present study an evaluation of the proposed digital musical instrument has been conducted. This evaluation has been conducted both from the audience and the performer perspective. According to the audience's evaluation responses, the EyeHarp digital music instrument offers a transparent correspondence between input gestures and the produced sound, i.e., participants in the study average rating of their understanding of the cause (cause comprehension), the effect (effect comprehension), and gesture-sound correspondence (mapping comprehension) was greater than 3.5 out of 5 (see Figure 8). This denotes a high level of transparency and comprehensibility in the actions and their relationship with the produced sound of the proposed music instrument. According to Hunt et al. (2002) and Arfib et al. (2005), these properties (transparency and comprehensibility) are positively correlated with the capacity of an instrument to allow the musician to produce expressive performances, and to engage the audience in the performances. Nevertheless, the obtained standard deviation for the gesture-sound correspondence (mapping comprehension) evaluation (*SD* = 1.33) indicates that some participants did not fully understand this correspondence. The standard deviation was smaller for the case of the cause and effect comprehension. Even though the EyeHarp being a diatonic DMI in which dissonant notes are very seldom produced, average audience evaluation of the error comprehension was high (i.e., 3.1). This again indicates a good understanding of the performer actions and corresponding produced music. All audience participants declared that they enjoyed the performance (average 4.3 out of 5). Most participants agreed that the interface allowed the performer to express his musical intentions (average 4.2 out of 5.0) which may be interpreted as an indication that the EyeHarp can allow the user to produce expressive performances.

Regarding the results of the evaluation from the performer's perspective, in the first task of the qualitative evaluation of the Step Sequencer Layer (i.e., the 8 × 8 grid task) it was achieved the best average time per selection. The resulting average time for the case of the 12 × 12 grid was almost double of the average time for the 8 × 8 grid task. This was expected, as small targets are harder to select. However, in the case of the 16 × 16 grid task the average selection time was less than the average for the 12 × 12 grid task. This can be explained by the fact that most of the notes in the 16 × 16 grid task were adjacent notes, which makes the visual search task easier.

The 8 × 8 grid arpeggio task can be compared to typical dwell-time eye-typing task, where the notes are replaced by characters. As seen in Figure 9, the average number of notes per minute in the 8 × 8 grid is close to the average number of characters per minute in dwell-time typing systems (17 chars/min according to Hansen et al., 2003).

In the case of the 8 × 8 grid the gaze feedback method produced the same results as the basic method, where the only visual feedback to the user is the brightening of the focus point at the center of the attended button. This result may be explained by considering the size of the 8 × 8 buttons: given their big size there was no difference with the two methods. On the contrary, in the case of the 12 × 12 and 16 × 16 grid, when the detected gaze coordinates were given as visual feedback, along with additional focus points, the performance (number of selected buttons per minute) increased with the gaze feedback method.

The experienced user participating in the study completed all the tasks of the step sequencer on average 2.8 times faster than the rest of the users. The difference is even higher in the case of the gaze feedback method. As concluded by Majaranta and Bulling (2014), if the user tries to look at the detected gaze coordinates, he may end up chasing the detected point, as it always is a few pixels away from the point he/she is looking at. It requires practice to learn how to take advantage of the visual feedback provided by the cursor in order to compensate for small calibration errors by adjusting the gaze point accordingly to bring the cursor onto an object. The experienced user clearly took more advantage of the gaze feedback than the non-experienced users. The difference in performance between the experienced user and the non-experienced ones may show that the EyeHarp is, similarly to traditional music instruments, an instrument in which practice play an important role.

The magnification method always performed worse than the gaze feedback method and only in the case of the 12 × 12 grid the obtained results were better than those obtained by the basic selection method. However, the magnification method always showed the lowest standard deviation on the number of selections per minute. This might explain why, as shown in Figure 11, the users show a preference for the magnification method over the gaze feedback method. The gaze feedback method might not be appropriate for novice users.

All in all, the evaluation of the step sequencer layer, confirmed all results reported by similar gaze controlled systems in which selecting targets using dwell-time selection method is required (Hansen et al., 2003; Majaranta and Räihä, 2007): (i) There is a steep learning curve in gaze interaction, (ii) magnification methods help in selecting small targets, and (iii) gaze visual feedback improves the performance of experienced users.

Figure 10 clearly shows that in the melody layer the experienced user (M32) achieves better temporal accuracy than any other user. This is an indication that there is a learning process involved for adapting to use the melody layer. Nevertheless, the number of accidental notes produced by the experienced user in tasks 2 and 3 are close to the average values across novice users. This indicates that the accidental notes are mainly caused by poor tracking accuracy, and not by the skill of the performer.

The number of omitted notes is higher in the tasks that require playing consecutively the same note (tasks 2 and 3). This is due to the behavior of the button responsible for note repetition: if a fixation is performed in the inner area of the pie but outside the “repeat note” button, the button disappears. In addition, due to noisy gaze tracking data, the user may be focusing on the center of the repeat button but the initial detected gaze point may fall outside the button area.

Although the tasks were designed with increasing difficulty, the average performance in the first task was similar to the average performance in the last task. This may be due to the training effect which compensates the different difficulty levels of the tasks. The last task is the most demanding, as it requires changing the chords along with the melody. A high number of accidental notes were observed during this task (as shown in Figure 10). This is due to he fact that the the positions of the chords and the notes are placed diametrically opposite in the interface.

The participants in the performer's perspective evaluation responded that the practice required to play the EyeHarp is comparable to the practice required to play a traditional musical instrument of average difficulty (3 out of 5 on average). The same response was given on average on the question about the the control the user has over the musical output (average value 3.1 out of 5), meaning that the control over the output is equivalent to that of a musical instrument that offers average control over the musical output.

The real-time feedback was rated high by most performers (average 3.9 out of 5). Most performers agree that playing music with the eyes is more tiring than playing with the hands (average 3.6 out of 5). Playing in tempo with the eyes is considered to be harder than playing with the hands (3.2 out of 5). Summarizing the above responses, we could conclude that performing music with the eyes is more difficult that performing with traditional means. Nevertheless, learning the EyeHarp gaze-controlled musical instrument wouldn't be harder than learning a traditional musical instrument.

The performer perspective evaluation was conducted with people with experience in playing musical instruments and no disabilities. In order to evaluate the EyeHarp in a more realistic setting, we would have required to test it with LIS patients. This, we believe, should be done in the future, and we have started looking for possible participants.

As summary, we have presented and evaluated the EyaHarp, a new gaze-controlled digital musical instrument. The system was evaluated from the performer and audience perspective. The obtained results indicate that, similarly to traditional music instruments, the proposed digital musical instrument allows to produce expressive performances both from the performer and audience perspective. The participants in the evaluation from the perspective of the performer responded that the practice required to master the EyeHarp DMI is similar to the average practice required to master a traditional musical instrument of average difficulty. The steep learning curve of the instrument is also reflected on the quantitative data, when comparing the performances of the experienced user with the novice users.

The cost of eye-tracking technology decreases every year. The last 5 years the cost of commercial eye-trackers has been reduced more than 10 times. Eye-tracking is slowly being incorporated in common place laptops, tablets and mobile phones. Such devices would allow many users, including users with motor disabilities, to have access to gaze-controlled applications, including the EyeHarp DMI.

The pEYE interface in the melody layer, provides a solution to the Mida's touch problem making it possible to play melodies in-tempo when the gaze of the user is used as the only input. If the physical abilities of the user allow it, other selection techniques like blinking, using physical buttons or blowing could be considered. If such selection methods were utilized, the user would be able to freely visually search the screen without triggering any undesired notes. This would allow increasing the number of available notes on the screen, as the central (neutral) area of the melody layer wouldn't be necessary. As future work, it would be interesting to compare the performance -in terms of overall usability, temporal accuracy and speed- of such an interface with the current version of the EyeHarp. The advantage of the screen button selection method may be that just one action is required to play a note: looking at the selection area. This might allow playing faster than in the case of using an independent clicking method which requires two actions (i.e., looking at the selection area and clicking). On the other hand, using an independent clicking method might allow placing more notes on the screen and might allow better temporal accuracy.

Probably the main target group of the proposed DMI is that of people diagnosed with Amyotrophic Lateral Sclerosis (ALS). ALS is a progressive neurodegenerative disease that affects nerve cells in the brain and the spinal cord. Individuals affected by the disorder may ultimately lose the ability to initiate and control all voluntary movements. Nevertheless, muscles responsible for eye movement are usually spared until the final stages of the disorder (Layer, 1953; Kiernan et al., 2011). A large number of studies have shown that music playing provides a variety of benefits (e.g., cognitive, psychological) (e.g., Hays and Minichiello, 2005). The EyeHarp DMI gives the opportunity to ALS patients to have access to such benefits. This could have a big positive impact in the quality of life of ALS patients -musicians or not-, by providing them the possibility of playing a musical instrument.

## Author contributions

ZV developed the software and technology presented in the paper and analyzed the experimental data. ZV and RR together designed the conducted the experiments and wrote the manuscript of the study.

## Funding

This project has received funding from the European Unions Horizon 2020 research and innovation program under grant agreement No. 688269, as well as from the Spanish TIN project TIMUL under grant agreement TIN2013-48152-C2-2-R.

### Conflict of interest statement

The authors declare that the research was conducted in the absence of any commercial or financial relationships that could be construed as a potential conflict of interest.

## Footnotes

^1^www.skoogmusic.com, last accessed 2015-4-12

^2^https://sites.google.com/site/windcontroller/, last accessed 2015-4-12

^3^http://illustriouscompany.co.uk/performance/duet-eyes-eyejamming-eyebodyweaving, last accessed on 10/12/2015

^4^http://sensorysoftware.com/, last accessed 2015-4-12

^5^http://www.tobii.com/, last accessed on 10/12/2015

^6^http://eyeplaythepiano.com/en/, last accessed on 10/12/2015

^7^http://www.getfove.com/, last accessed on 10/12/2015

^8^http://www.openframeworks.cc/, last accessed on 14/12/2015

^9^Source code and binaries available at https://github.com/zackbam/TheEyeHarp

^10^https://theeyetribe.com, Last accessed on 17/12/2015

^11^http://www.tobiidynavox.com/pceye-go/, Last accessed on 17/12/2015

^12^http://sourceforge.net/projects/gazetrackinglib/, Last accessed on 17/12/2015

^13^http://www.nerds.de/en/loopbe1.html, last accessed on 17/12/2015

^14^Available online at https://youtu.be/dS5QkIgK0NY

^15^http://www.reaper.fm/, last accessed on 16/3/2015
